# Community service rehabilitation therapists’ understanding of social accountability

**DOI:** 10.4102/phcfm.v16i1.4473

**Published:** 2024-06-06

**Authors:** Ntandoyenkosi L. Msomi, Andrew J. Ross

**Affiliations:** 1 Department of Family Medicine, College of Health Sciences, University of KwaZulu-Natal, Durban, South Africa

**Keywords:** social accountability, understanding, rehabilitation therapists, community-based rehabilitation, healthcare

## Abstract

**Background:**

Social accountability is the obligation of health care providers to address the priority health concerns of the community they serve and of universities to ensure that graduates understand these social responsibilities. Although social accountability can combat systemic health inefficiencies, it is not well-understood or practised.

**Aim:**

The study aimed to explore community service rehabilitation therapists’ understanding of social accountability.

**Setting:**

The study was conducted in KwaZulu-Natal, South Africa.

**Methods:**

This study used an interpretive exploratory design and purposively recruited 27 community service rehabilitation therapists namely, audiologists, speech-language therapists, occupational therapists, and physiotherapists working in public sector health facilities in rural and peri-urban areas. Four focus group discussions and four free attitude interviews were conducted, the results being thematically analysed.

**Results:**

Despite most of the participants not being instructed in social accountability as part of their formal training or institutional induction, three themes emerged based on their experiences. These themes include describing social accountability, values of social accountability, and values of community-based rehabilitation applicable to social accountability.

**Conclusion:**

Inclusion of instruction on social accountability as part of their formal training and health facility induction would contribute to rehabilitation therapists’ understanding of social accountability.

**Contribution:**

The study contributes to data on rehabilitation education and community service training regarding social accountability within a South African context and has captured how experiences gained during community service contribute to the rehabilitation therapists’ understanding of social accountability.

## Introduction

Rehabilitation services encompass the prevention, promotion, assessment, and treatment of persons with disabilities and those at risk of developing disabilities. In South Africa (SA), all rehabilitation health care graduates are required to undertake 1 year of community service in the public health care sector before obtaining full registration with the Health Professions Council of South Africa (HPCSA). This entails working at primary healthcare clinics, district, and regional hospitals, and conducting outreach events, primarily catering for those who cannot access health care. Rehabilitation therapists involved in patient care include the disciplines of audiology, speech-language therapy, occupational therapy, and physiotherapy with many district hospitals and clinics in SA being largely staffed by those cadres in their community service year. This means that rehabilitation therapists working with a vulnerable section of the population are young and relatively inexperienced. They may also not have the opportunity to form an idea of social accountability by years of experience in the field.

The South African healthcare system is divided into public and private sectors, with 71% of its citizens accessing public services and 27% accessing private care.^1^ In the province of KwaZulu-Natal (KZN), 79% of the 11.5 million inhabitants use the public sector and 27% use private healthcare.^2^ However, the South African health review report of 2020 indicated an inadequate therapist to patient ratio in the public sector with only 3.10 physiotherapists (PTs), 2.64 occupational therapists (OTs), and 1.51 speech therapists and audiologists (STAs) per 1000 people respectively.^1^ In 2018, 74% of OTs worked in the private sector and only 16% of OTs served in the public sector.^1^ This inequitable distribution of therapists was further exacerbated by the impact of the coronavirus disease 2019 (COVID-19) pandemic which disrupted rehabilitation services, particularly in the public sector.^3^

The shortage of rehabilitation therapists in SA poses a significant barrier to delivering rehabilitation services, and hinders efforts to extend access to these services to the general population.^3^ Globally, the United Nations reports that 15% of the world’s population has a disability, with 80% of those with disabilities residing in low- and middle-income countries.^4^ Many people with disabilities live in poverty in relatively rural contexts, which also widens the barrier to rehabilitation services as fewer rehabilitation professionals are working in rural than in urban areas.^5^ The disability and development report highlighted that in the Southern African region, 64% of people with disabilities are not able to access rehabilitation services.^6^ With disability rates ranging from 4.4% – 16.1% in SA, the need for rehabilitation therapists far exceeds the current capacity of those working in the public sector, with data showing that this is worse in rural areas.^7,8^

To address the challenges of providing access to rehabilitation services in rural and low resourced settings, the World Health Organization (WHO) proposed the implementation of a community-based rehabilitation (CBR) approach. This approach aims to ensure equal access, at a primary health care level, to good quality service for persons with disability, mainly focusing on prevention and promotion.^9^ Community-based rehabilitation addresses five key elements, namely health, education, livelihood, socialisation, and empowerment, ultimately aiming to enhance the quality of life of people with disabilities.^10^ The health element of the CBR matrix includes components, namely promotion, prevention, medical care, rehabilitation and assistive devices.^10^ Rehabilitation is thus a component of the health domain of CBR, which focuses on modification of impairments, compensation for loss of function, and modification of the environment.^4^ Rehabilitation therapists working in CBR offer more services beyond rehabilitation. This may include advocating for the needs of wheelchair users with the local transport department and conducting health promotional campaigns in the community.

Within this context, the WHO has highlighted the importance of social accountability which is:

[*T*]he obligation of the faculty to direct their education, research, and service activities towards addressing the priority health concerns of the community, the region, or the nation they have the mandate to serve.^11^

This obligation starts by understanding existing community healthcare needs and thereafter addressing them. The WHO definition of social accountability has been largely adopted by higher education institutions and it has thus been incorporated into health sciences curricula development. This has led to an emphasis on primary healthcare and community-based education, clinical placements of students in underserved communities, and recruiting students who complement the profile of the community.^12^ Although there is no universal definition of social accountability, two elements are generally seen in many definitions, that is, ‘provider’ and ‘recipient’.^13^ Social accountability has been proposed as the appropriate guide for the practice of medicine and the delivery of healthcare services in urban and rural contexts.^14^ Acquisition of social accountability awareness, technical skills needed to address issues, and professional identity are interlinked and essential to improve healthcare services.^15^ Wollard delineated a partnership pentagram of social accountability that included academic institutions, communities, healthcare professionals, health administrators, and policy makers.^16^ The partnership pentagram of social accountability depicted equally weighted roles across the five partners and further how these partners can transform an existing healthcare system to be more responsive to current community needs. Social accountability and CBR both emphasise management of community’s healthcare needs. This requires services beyond rehabilitation such as intersectoral collaboration with health district coordinators, consultations with institutions training healthcare professionals, and implementation of support groups for the various community groups requiring their expertise. While extensive research has been conducted on social accountability in medical practice and education, using the WHO definition of social accountability,^11^ little attention has been given to understanding of social accountability from the perspective of rehabilitation professionals.^17^ In their study, Doja and colleagues emphasised that people’s perceptions of social accountability are influenced by a variety of factors, including a lack of clinical and administrative supervision, restricted access to information, a lack of transparency among community members, and the unwillingness of service providers to offer services.^18^ The study seeks to contribute to the existing body of knowledge by exploring community service rehabilitation therapists’ understanding of social accountability in KZN province, SA.

## Research methods and design

### Study design

The study employed a qualitative approach and an interpretive exploratory research design to explore rehabilitation therapists’ understanding of social accountability. The study design allowed rehabilitation therapists to contribute to the development of new unique information regarding their understanding of social accountability during community service training.^19^ The study was concluded in October 2023, the participants having completed 10 months of their community service year.

### Study population and sampling strategy

The target population for this study were community service audiologists (AUDs), occupational therapists (OTs), speech-language therapists (SLTs) and physiotherapists (PTs) involved in CBR. To be included in the study, they had to meet the following criteria. Firstly, they had to be registered with HPCSA in the community service officer category in one of the four designated professions, and secondly, they had to be involved in a CBR environment. The population size was 212 community service rehabilitation therapists working in KwaZulu-Natal. The size of the sample frame was 136 community service rehabilitation therapists working in the eThekwini, King Cetshwayo, Zululand, iLembe, Harry Gwala, and Ugu districts. The number of rehabilitation therapists sampled and approached were 30, as this was deemed dependable based on the principles of Malterud and colleagues.^20^ Twenty-seven community service rehabilitation therapists consented and participated in the study.

Purposive sampling with the maximum variation strategy was used to recruit participants. The criteria included recruiting a fair representation of rehabilitation therapists across the four disciplines and also from those working in hospitals and primary healthcare clinics. Requests for participant letters were emailed to the Departments of Audiology and Speech Therapy, Occupational Therapy, and Physiotherapy within the six designated districts.

### Data collection

A combination of four focus group discussions and four free attitude interviews were utilised for this study and data were collected virtually via Zoom (Zoom Video Communication, San Jose, California, United States). The interview guide consisted of three sections; firstly, their demographics as can be seen in Table 1 (e.g. age, gender, training institutions), secondly, open-included questions on their understanding of social accountability and CBR, and thirdly, the challenges they had experienced. The audio-recorded focus group discussions and free attitude interviews lasted 30 min – 90 min and were facilitated by the researcher.

**TABLE 1:** Demographic details (*N* = 27).

Code	Age	Gender	Discipline	University	Location
FAIP1	23	Male	PT	UKZN	Peri-urban
FAIP2	23	Male	AUD	UKZN	Rural
FAIP3	22	Female	SLT	UKZN	Peri-urban
FAIP4	23	Female	OT	UP	Rural
FG1P1	23	Female	OT	UCT	Peri-urban
FG1P2	23	Female	PT	UKZN	Peri-urban
FG1P3	25	Male	SLT	UKZN	Rural
FG1P4	24	Female	PT	UP	Peri-urban
FG1P5	25	Female	AUD	UCT	Peri-urban
FG2P1	24	Female	PT	UKZN	Rural
FG2P2	23	Female	OT	UKZN	Peri-urban
FG2P3	22	Female	AUD	UKZN	Rural
FG2P4	23	Female	SLT	UKZN	Peri-urban
FG2P5	24	Male	SLT	UKZN	Rural
FG2P6	23	Female	AUD	UKZN	Rural
FG2P7	23	Female	OT	UKZN	Peri-urban
FG3P1	25	Female	AUD	UKZN	Rural
FG3P2	24	Male	OT	UP	Peri-urban
FG3P3	22	Female	SLT	UKZN	Rural
FG3P4	28	Female	SLT	UKZN	Rural
FG3P5	27	Female	PT	UCT	Peri-urban
FG4P1	23	Female	AUD	UKZN	Rural
FG4P2	22	Female	PT	UKZN	Rural
FG4P3	24	Female	SLT	UKZN	Rural
FG4P4	23	Female	SLT	UKZN	Rural
FG4P5	22	Female	OT	UKZN	Rural
FG4P6	24	Female	SLT	UKZN	Rural

PT, physiotherapist; AUD, audiologist; SLT, speech-language therapist; OT, occupational therapist; UKZN, University of KwaZulu-Natal; UP, University of Pretoria; UCT, University of Cape Town.

### Data analysis

The interviews were transcribed verbatim; transcripts were imported into NVivo 12 software for thematic analysis.^21^ The researcher and an independent qualitative coder assessed cross-group saturation by comparison analysis with saturation being reached when no new themes were derived. The researcher and the independent coder repeatedly read the transcripts to become familiar with the data. Thereafter a coding framework was developed, and applied to all transcripts that were coded separately. Coding was conducted line by line to formulate free codes that were later grouped to form themes and thereafter categories. A consensus meeting was used to discuss any discrepancy in identified codes, until a final list of codes and themes was derived.

### Ethical considerations

Ethical approval was provided by the Humanities and Social Sciences Research Ethics Committee (HSSREC/00005538/2023) of the University of KwaZulu-Natal. The KwaZulu-Natal Department of Health Research Committee (KZ_202305_030) approved the study with the Deputy Director of Disability and Rehabilitation Services providing gatekeeper permission, while written informed consent was obtained from the participants.

## Results

Of the 30 who were invited to participate, 27 agreed, of whom 22 were female with their ages ranging from 22 to 30 years, with five working in peri-urban and 22 in rural areas. Two participants obtained a BSc (Hons) in Physiotherapy and a BA in Speech-Language Pathology as a second qualification.

Table 2 shows the themes that emerged and the associated sub-themes. The meaning of social accountability generated several responses and definitions and will now be discussed in the following section. To ensure anonymity, each participant was allocated a unique code, with those in focus group discussions being indicated as focus group participant (FGP), and in the free attitude interviews as free attitude interview participant (FAIP).

**TABLE 2:** Key themes identified from the data.

Key themes	Sub-themes
**1. Describing social accountability**	1.1. Defining social accountability 1.2. Wise resource use 1.3. Respond to needs 1.4. Serve the community 1.5. Community involvement
**2. Values of social accountability**	2.1. Respect 2.2. Compassion and empathy 2.3. Creativity and adaptability
**3. Values of CBR which can be applied to social accountability**	3.1. Goal oriented 3.2. Adaptability 3.3. Collaboration

CBR, community-based rehabilitation.

### Theme 1: Describing social accountability

Five sub-themes emerged from the group discussions and interviews, these being: defining social accountability, wise resource use, respond to need, serve the community, and community involvement.

#### Sub-theme 1.1: Defining social accountability

Very few participants had heard of social accountability or knew what it meant, as indicated by their responses:

‘I do not know the term.’ (FG1P2, Female, PT)

‘I do not quite understand it.’ (FG4P3, Female, SLT)

‘I do not have an idea, but I can try.’ (FAIP4, Female, OT)

‘I do not know the book definition of what social accountability is.’ (FAIP2, Male, AUD)

Most indicated that while this subject was never taught to them during their undergraduate years, they were able to draw lessons from their training and experiences, which they now understood as aspects of social accountability, this being a complex concept:

‘I do not remember being directly taught about social accountability.’ (FG2P5, MALE, SLT)

‘… [*T*]hese things are not directly taught, you learn through experience.’ (FAIP2, Male, AUD)

‘… [*T*]here is a minor connection to what we learned in school and how we practice social accountability post-school.’ (FG2P7, Female, OT)

‘… I agree with the other participants that it was more of something that was implied than, you know, directly taught and directly explained.’ (FG2P3, Female, AUD)

#### Sub-theme 1.2: Wise resource use

In their attempt to define social accountability, most participants re-used the words ‘accountability’ and ‘responsibility’, described it as a form of accountability, that being an obligation or willingness to accept responsibility or to be accountable for one’s actions as part of professional ethics. As professionals, and being paid for their services, they needed to ensure that they maximised the use of the available resources, they provide the required services, ensure high-quality work, and maximise their time.

This need to optimise the use of resources to improve the quality of life and health of the community was shared among most of the participants:

‘The services we provide should be accountable regarding the resources and how they are delivered to the community.’ (FAIP1, Male, PT)

‘… [*T*]he responsibility you have as a healthcare professional to account for how you use the resources in the hospitals to improve the patient’s quality of life and make sure that they get the healthcare that they deserve … being transparent in everything we do with our patients.’ (FG2P4, Female, SLT)

This transparency and accountability for some participants extended to how they use their time effectively in serving the community. The participant referenced instances where the therapists had no referrals and suggested that this time could be used creatively and productively towards CBR and should not be wasted because there were always needs to be met:

‘… [*I*]s not stealing state time and using whatever free time to fill in the gaps in your department.’ (FG2P3, Female, AUD)

For other participants, social accountability concerns the quality of work and services rendered and demands high levels of professionalism at all times from healthcare professionals. This commitment and dedication to quality service is not limited to the working environment but also extends to the services rendered to the community. Most participants highlighted the need to be responsible and accountable for treatment as part of social accountability:

‘As healthcare professionals, we must be accountable for our work quality … accountability is providing your best and fulfilling the community’s needs.’ (FG4P1, Female, AUD)

‘I feel that as a healthcare professional, you must do your best to the community … So, in your professional life, I feel like the people you service and where you work, you should give 100%.’ (FG4P2, Female, PT)

‘So, when we conduct home visits, it is essential also to do reviews or treatment follow-ups to show accountability in your management plans. Being socially accountable in the CBR context is like looking at what works for the patient and not just leaving therapy hanging.’ (FG1P1, Female, OT)

The participants perceived that the onus of being socially accountable was placed on the individual and required them to take responsibility for their actions.

#### Sub-theme 1.3: Responding to needs

Most of the participants defined social accountability as responding to and meeting the needs of the community, first of all, by identifying them and then taking appropriate action to address them and doing so consistently.

‘… [*S*]ocial accountability is community needs analysis and the management thereof.’ (FG2P6, Female, AUD)

They gave examples of being socially accountable as assisting a patient to get the correct wheelchair after being given one that was too small and providing a cushion to help ease the pressure off their legs. Another shared about how they had started literacy drives in the community to help children with reading and writing difficulties:

‘We were not obliged to go to the schools, but as part of being accountable in society, we saw a problem, and we tried to think, how can we fix it?.’ (FG2P5, Male, SLT)

The results show that participants believe being socially accountable is tied to meeting the community’s needs and demands.

#### Sub-theme 1.4: Serving the community

Many participants regarded social accountability as being the responsibility and obligation of providing quality and professional services to the communities they served. This includes service delivery being a priority, being centred on treating the community with dignity, and Ubuntu. This view was shared by most of the participants, who practise in African communities:

‘What comes to mind when I think about social accountability has to be recognising that everybody is a person because of other people, so basically Ubuntu.’ (FG3P3, Female, SLT)

‘The community comes first, so you must try to provide services that are convenient for them.’ (FG2P2, Male, OT)

Most participants felt that it is essential to understand the context and cultures of the community to treat patients as dignified human beings. One aspect of treating people with dignity was the need for healthcare professionals to adopt a more human approach to addressing their patients:

‘Firstly, as a healthcare professional, when referring to patients, making sure you are not referring to them as the Down syndrome child, but the child who has Down syndrome … it is not diagnosis first, but person first terminology.’ (FG1P4, Female, PT)

#### Sub-theme 1.5: Community involvement

Some participants viewed social accountability in terms of community involvement, being rooted in the relationship between the healthcare professionals and the people they serve. This involvement of the community being served is critical for achieving effective CBR and contributes to the relevance and success of any CBR initiative:

‘It is like a relationship between you and the people you are going to help.’ (FG1P2, Female, PT)

‘It is more about involving your people, patients or community.’ (FG1P5, Female, AUD)

‘[*A*]llows people to voice their needs.’ (FG1P1, Female, OT)

‘… [*M*]akes them part of the decision-making process.’ (FG1P2, Female, PT)

For some participants, this community involvement entails the fact that the community’s voices, perspectives, and concerns are brought to light and can be attended to. One participant recalled an incident in which a patient caused a scene at the hospital and lodged a complaint to the CEO regarding the lack of services that she had received:

‘By complaining, she was accountable, and made the work easier for us to attend to the matter.’ (FG3P4, Female, SLT)

By voicing their concerns, the community members are also socially accountable and responsible for their own health management. This entailed taking some responsibility for their development, being active and responsible participants in the CBR process, and protecting their shared resources as a community, as noted by a participant:

‘They must be accountable for protecting the clinic against robbery and vandalism.’ (FG4P3, Female, SLT)

Social accountability was therefore regarded as a responsibility shared by healthcare professionals and the community they serve.

### Theme 2: Principles of social accountability

Despite the lack of education on the concept, the participants shared the qualities that they felt were required to ensure social accountability, these being respect, compassion and sympathy, and creativity.

#### Sub-theme 2.1: Respect

Respect was regarded as a crucial aspect of social accountability, particularly in a culturally diverse context with different belief systems and values, with healthcare professionals being required to be aware of such dynamics and able to conduct themselves respectfully. While social accountability was not taught as a subject during their training, many participants referred to the Community module in undergraduate training as providing relevant instruction:

‘You need to be respectful to the context and cultural appropriateness of the patients.’ (FAIP1, Male, PT)

‘In the Community module, we learn CBR and Batho Pele principles.’ (FG1P2, Female, PT)

#### Sub-theme 2.2: Compassion and empathy

Being compassionate and showing empathy was also regarded as being important for achieving social accountability by community service rehabilitation therapists:

‘To have that sort of compassion and empathy for people to display social accountability in terms of your workspace.’ (FAIP2, Male, AUD)

‘One aspect I learned was to be compassionate and empathetic towards patients, as we deal with people that have disabilities and are most probably already being stigmatized by society.’ (FG1P5, Female, AUD)

The participants felt that these attributes came naturally, given that their professions were those that assisted people to live more independently and have a better quality of life.

#### Sub-theme 2.3: Creativity and adaptability

The participants noted the importance of being creative in finding ways to assist their patients, and that while their theoretical training provided a useful foundation, they needed to use their initiative to find solutions. This was particularly relevant in the public health sector, where resources were sometimes limited. Others used the terms ‘adaptable’, ‘improvise’, ‘tailor’, and ‘innovative’ to describe what they learned. One participant noted that:

‘Yeah, being adaptable is important due to working with limited resources … we had to think about what they would need and what would be beneficial to them, because just doing something that would not be sustainable is not going to help the community in any. So, I learned to tailor whatever I do to the patient, based on cultural and linguistic aspects to suit the patient. This ensured that assessment and treatment results were valid and reliable.’ (FG1P4, Female, PT)

The participants acknowledged that there is a great need for the health care professional to be creative, considering that they practise in situations that are characterised by challenges that may be context-specific.

### Theme 3: Values of community-based rehabilitation applicable to social accountability

Most participants understood social accountability and CBR as being closely interdependent and were ‘interlinked’, ‘go hand-in-hand’, ‘inter-related’, ‘linked’, and ‘inseparable.’ They regarded the value of social accountability as being in its relationship with CBR. This is indicated in the sub-themes that emerged of helps to achieve goals, adaptability in addressing priority health concerns and promotes collaboration.

#### Sub-theme 3.1: Helps to achieve goals

Most participants regard social accountability as valuable as it helps to achieve CBR’s goals where they are needed and by whom, as supported by other participants in the focus group discussions:

‘Social accountability means constantly asking ‘is my service meeting the needs of the community?’ and CBR is part of the answer.’ (FAIP4, Female, OT)

‘Social accountability underpins one of the main principles of CBR, which is to make sure that it is carried out effectively and where it should be carried out.’ (FG1P4, Female, PT)

The participants agreed that social accountability is of great value in accomplishing effective CBR, without which healthcare professionals would be doing their patients, and therefore the community, a disservice:

‘In CBR, social accountability is highly valued because you are accountable for all decisions. Without that sense of accountability, you are doing a disservice to patients.’ (FG3P3, Female, SLT)

#### Sub-theme 3.2: Adaptability in addressing priority health concerns

An important key value of CBR, as it relates to social accountability, was noted as being its ability to address the priority health issues identified by the communities being served, not only by the professionals. This was a view shared by most of the participants, and was captured in the response of a participant in the focus group discussion:

‘It is a very important factor to look at in community-based rehabilitation, because to me it is where you address concerns, so being socially accountable, you can go out and address priority health concerns of the community … you will not be effective if you target things that are not a problem in that certain area.’ (FG1P2, Female, PT)

Engaging in social accountability was connected to identifying the needs and responding to the problems identified, which required the health professional to have a sense of adaptability regarding their skills and resources to ensure that they offered appropriate services to particular groups of patients:

‘You must identify the community’s needs and tailor your services accordingly.’ (FG2P7, Female, OT)

‘… [*U*]sing a one size fits all approach is not practical and unethical.’ (FG4P2, Female, PT)

Thus, social accountability allows health professionals to offer person-centred care, which is crucial in CBR.

#### Sub-theme 3.3: Collaboration

Social accountability is important in CBR as it improves collaboration between healthcare professionals and their community, who also need to take responsibility for making it successful, requiring both service providers and recipients to be involved in decision-making. When the community takes some responsibility for social accountability, it assists the healthcare professionals to render services more easily as it becomes collaborative, as indicated in the comments:

‘We are responsible for service delivery, but the community also has responsibility for certain things; this makes rendering services easy for both parties.’ (FG3P4, Female, SLT)

‘I do not think you can have a good successful CBR without the society being accountable for their developments and services that they get.’ (FG4P3, Female, SLT)

Collaboration and engagement also pave the way for better communication between the stakeholders, which leads to improved service delivery.

## Discussion

Regarding awareness of social accountability, most participants had either not heard about the concept or lacked a clear understanding of the concept making it challenging for them to provide a description of it. Notably, participants had not received instruction on social accountability during their studies at any of the South African universities from which they had graduated from. This lack of awareness was consistent with studies conducted in Morocco and India, where only one-third (38.4%) of undergraduate students had a clear understanding of social accountability.^22^ Although participants in this study were recent graduates, their awareness of social accountability appeared to be based on experiences during their community service rather than in the practices acquired during their undergraduate training, which lacked teaching on the issue, a trend observed in similar studies.^23,24^ Most studies on social accountability have been conducted with students in Asia,^22^ Latin America,^25^ the Eastern Mediterranean,^26^ and a growing number of studies from Africa.^26,27^ These studies have consistently shown a lack of knowledge of social accountability among final year health sciences students,^10,28^ and a need to explore how to bridge the gap between theoretical instruction and health practice to embrace concepts of social accountability.

Despite participants’ inability to clearly define the concept, because of their experiences during community service, they associated it with the wise use of resources, responsiveness to the community needs, and active community involvement. These findings align with other studies, where healthcare professionals linked human dignity, responsiveness, compassion, and respect to their understanding of social accountability.^29^ The literature shows that most scholarly publications interchangeably use responsibility, responsiveness, and accountability.^13^

Participants showed reflection when they emphasised accountability for the wise use of resources including their time, as they generally share working space and need to account for resources taken from the hospitals to the community. Resources are accounted for using an asset register at the base hospital and reviewed quarterly to ensure timeous procurement of resources for therapy. Other participants regarded social accountability as achieving Ubuntu with community members, a traditional African concept emphasising humanity towards others, which conveys awareness of other people’s needs and beliefs, and that current circumstances reflect the past and a consequence for the future.^27^ This perspective aligns with studies indicating that the involvement of clinicians in community projects was regarded by community members as showing Ubuntu and being socially accountable.^24^ Effective social accountability, as suggested by the participants, is best accomplished when healthcare professionals embrace values such as respect for human dignity and Ubuntu. This finding aligns with the literature, which highlights the fact that effective social accountability is possible only when healthcare professionals internalise humanity and ethics.^28^ The participants in this study revealed that healthcare professionals need to exhibit an understanding for humanity, as this is crucial to achieving social accountability.

The participants’ emphasis on responsiveness to community needs is in line with other studies that have highlighted the value of commitment and concern for human life, as well as advocating for actions that positively affect the lives of others.^26^ Empowerment was also central to the participants’ understanding of social accountability, and while the participants did not use the term, community involvement is a critical component of empowerment within the CBR matrix.^30^ The objectives of CBR empowerment aim to achieve informed consent, involve the community in the decision-making processes, and mobilise them to deliver services and resources.^30^ Bodang in her study describes empowerment as enabling recipients of CBR services to become ‘people in the full sense of the word’ by providing equal opportunities, fulfilment and satisfaction, improved quality of life, and greater involvement in community projects.^31^ The participants regarded respect as a relevant aspect of social accountability to achieve cultural appropriateness. Respect is generally centred around patient autonomy, although the participants broadened the view of contextualised services, to include cultural appropriateness and achievement of Batho–Pele principles. The perspectives of respect were mutual across the participants from different ethnic groups.

Compassion and empathy were highlighted as key aspects of social accountability which contributed to improved quality-of-service delivery particularly in the context of serving a patient population with disabilities, which often reduces their employment opportunities and thus leads to an impoverished lifestyle. While the terms *empathy* and *compassion* cannot be used interchangeably, both strengthened the therapist–patient relationship and contributed to regular attendance at consultations. A factor that influenced therapists to be empathetic and compassionate towards patients in rehabilitation was engagement with those who were stigmatised by society and vulnerable to misinformation. Khoza highlights the fact that respect, compassion, and empathy are African humanism components that are interrelated and essential for a transformative working relationship such as that of a patient to therapist.^32^

Creativity and adaptability were identified as skills learnt during undergraduate training and deemed necessary to practise rehabilitation and social accountability. Although the gazetted scope of practice for audiology, speech-language therapy, and occupational therapy documents cultural adaptations as a requirement for patient management, participants highlighted the ability to adapt because of resource constraints as an additional, important skill.^33,34,35^ Creativity and adaptability contribute to individualised, culturally sensitive, and innovative management plans for patients. This aligns with research which emphasises the symbiotic relationship between creativity and social responsibility, potentially improving service delivery, the wellbeing of patients, and adherence to management plans given by healthcare professionals.^36^ Participants felt that the goal orientation focus of rehabilitation, which was guided by their patient needs, helped them to be more patient-centred and socially accountable. Collaboration among relevant stakeholders, particularly between community members and rehabilitation therapists was considered crucial for effective social accountability in rehabilitation. However, participants did not address challenges or consequences when collaboration is not achieved.

### Limitations

A number of limitations may have affected the findings, including the fact that therapists were not included from urban areas, that only a few sites in KZN were included, most were rural areas. Despite the limitations, the consistency of the themes that emerged among the different disciplines suggests that they all had similar experiences, and that their training had sensitised them to the need for community inclusivity and cultural appreciation. The results showed that community service therapists had an awareness of the concept of social accountability and the need to practise social accountability. In addition, the results further show that rehabilitation therapists consider the principles of CBR transferable and applicable to social accountability practice. A mixed methods approach would strengthen the study as the sample may be widened by including a quantitative aspect.

### Recommendations

The following recommendations arise out of this study:

- All disciplines that are required to undertake a year of community service should receive explicit instruction on social accountability as part of their preparation for working in the healthcare sector.
- Workshops about the importance of social accountability in determining the services provided by community service rehabilitation therapists should be provided in all facilities.
- The understanding of rehabilitation therapists towards social accountability and how this affects their attitudes and practices in different settings needs further investigation.

## Conclusion

Although not formally covered during their training, community service rehabilitation therapists have developed a good understanding of social accountability through their personal experiences while working as rehabilitation therapists. This understanding has contributed to a greater awareness of the broader social responsibility associated with serving those with disabilities.
